# Correction to “Developing an optimal maternal‐fetal medicine ultrasound practice: A report and recommendations of the workshop of the Society for Maternal‐Fetal Medicine”

**DOI:** 10.1002/pmf2.70062

**Published:** 2025-06-25

Society for Maternal‐Fetal Medicine (SMFM), Joanne Stone, Bryaan Bromley, Mary E. Norton, Helen Feltovich, Lawrence D. Platt, Joshua A. Copel, et al. 2025. “Developing an optimal maternal‐fetal medicine ultrasound practice: A report and recommendations of the workshop of the Society for Maternal‐Fetal Medicine.” *Pregnancy* 1(3): e70003.

In the originally published version of this article, Juliana G. Martins’ name was omitted, and Jean L. Spitz's name was misspelled in the author byline. The correct byline should have read:

Society for Maternal‐Fetal Medicine (SMFM), Joanne Stone, Bryann Bromley, Mary E. Norton, Helen Feltovich, Lawrence D. Platt, Joshua A. Copel, Reem S. Abu‐Rustum, Mishella Perez, Katherine Connolly, Juliana G. Martins, Martha W. Rac, Anne H. Mardy, Therese Cooper, Yinka Oyelese, Arij Faksh, Thomas Lee, Shad Deering, Jean L. Spitz, Alfred Abuhamad.

We apologize for these errors.

